# Real-Time Path Planning Based on Harmonic Functions under a Proper Generalized Decomposition-Based Framework

**DOI:** 10.3390/s21123943

**Published:** 2021-06-08

**Authors:** Nicolas Montés, Francisco Chinesta, Marta C. Mora, Antonio Falcó, Lucia Hilario, Nuria Rosillo, Enrique Nadal

**Affiliations:** 1 Department of Mathematics, Physics and Technological Sciences, University CEU Cardenal Herrera, C/San Bartolome 55, CP Alfara del Patriarca, 46115 Valencia, Spain; afalco@uchceu.es (A.F.); luciah@uchceu.es (L.H.); nrosillo@uchceu.es (N.R.); 2 PIMM Lab, ESI Group Chair at ENSAM Institute of Technology, 151 Boulevard de L’Hôpital, 75013 Paris, France; francisco.chinesta@ensam.eu; 3 Department of Mechanical Engineering and Construction, Universitat Jaume I, 12071 Castellón, Spain; mmora@uji.es; 4 Centro de Investigación en Ingeniería Mecánica, Universitat Politécnica de València, 46022 Valencia, Spain; ennaso@upvnet.upv.es

**Keywords:** path planning, potential fields, harmonic functions, Proper Generalized Decomposition, Poisson equation

## Abstract

This paper presents a real-time global path planning method for mobile robots using harmonic functions, such as the Poisson equation, based on the Proper Generalized Decomposition (PGD) of these functions. The main property of the proposed technique is that the computational cost is negligible in real-time, even if the robot is disturbed or the goal is changed. The main idea of the method is the off-line generation, for a given environment, of the whole set of paths from any start and goal configurations of a mobile robot, namely the computational vademecum, derived from a harmonic potential field in order to use it on-line for decision-making purposes. Up until now, the resolution of the Laplace or Poisson equations has been based on traditional numerical techniques unfeasible for real-time calculation. This drawback has prevented the extensive use of harmonic functions in autonomous navigation, despite their powerful properties. The numerical technique that reverses this situation is the Proper Generalized Decomposition. To demonstrate and validate the properties of the PGD-vademecum in a potential-guided path planning framework, both real and simulated implementations have been developed. Simulated scenarios, such as an L-Shaped corridor and a benchmark bug trap, are used, and a real navigation of a LEGO^®^MINDSTORMS robot running in static environments with variable start and goal configurations is shown. This device has been selected due to its computational and memory-restricted capabilities, and it is a good example of how its properties could help the development of social robots.

## 1. Introduction

A fundamental robotic task is to plan collision-free motions among a set of static and known obstacles from a start to a goal position. The geometric construction of this planning strategy is computationally hard and hence unfeasible for its use in real-time (RT) applications [1] where the environment changes, as is the case of social robotics. This motion planning (or the piano mover’s) problem has motivated many works in the field of robotics, and its complexities are well known in the literature. The problem cannot be solved in closed form and, therefore, approximations and simplifications have been developed to partially solve the problem. Some researchers have been devoted to study some sub-classes of the general problem while other researchers have considered alternative and simplified planning paradigms such as sampling-based planners, grid-based and interval-based searches, combinatorial methods, and potential-field-based techniques [2,3]—all of them attempting to find a trade-off between completeness and computational burden.

In the above context, one of the most popular algorithms is the so-called Artificial Potential Field technique (APF) [1,4,5]. This method introduces an artificial potential field that produces a set of paths from a start to a goal configuration. It allows the computation of a unique trajectory from the start to the goal. This technique is very fast for RT applications, except when the vehicle is trapped in a deadlock (a local minimum of the potential function). For this reason, this technique is currently being investigated in the fields of mobile robotics [6], intelligent vehicles [7,8] and social robotics [9,10,11].

The solution to this problem lies in the use of harmonic functions to generate the potential field [12]. These functions, initially proposed in [13], appear as the solutions of the Laplace equation on a specific domain. They exhibit very elegant and interesting properties for path planning, as described in [14]. First, harmonic functions satisfy the min–max principle and, therefore, the appearance of deadlocks is not possible. Second, under certain assumptions, a path planning scheme using harmonic functions is complete. Third, solutions to Laplace’s equation obey the principle of superposition, which can be used to enhance the vehicle behaviour in certain areas of the workspace (near the obstacles). Fourth, the gradient of a harmonic function can be used as a velocity reference for the mobile robot navigation. Finally, harmonic-based navigation allows dealing with errors in the model and in the mobile robot position, i.e., with uncertainty.

Despite their attractive properties, path planning based on harmonic functions has not been widely adopted because these functions cannot be computed in closed form and discrete approximations imply such a computational burden that has prevented their extensive use, as indicated in [15]. In fact, standard finite difference methods (such as Jacobi, Gauss–Seidel, SOR) are typically used to solve the Laplace equation [14,16], implying the use of static environments, i.e., static obstacles as well as fixed start and goal configurations. Any new configuration requires the recalculation of the harmonic function and the computation of the streamlines over the entire region. Only if everything remains static, path generation can proceed very quickly since it only involves the evaluation of the gradient of a precomputed potential function. Additionally, as explained in [17], the solution must be numerically obtained in a discrete mesh, which entails a computational cost that increases exponentially with the mesh resolution. For instance, in [18], a scanned environment composed of 1500 triangles was used that implied a computational cost of 19.2 s in an Intel Core 2 Processor (64-bit dual-core commercial CPU) for every re-computation of the streamlines. Although recent techniques have accelerated this calculation [19,20], the computational time is still high for RT navigation, with 646s for a 512 × 512 node environment using the EGSOR algorithm in the last work. Thus, its use for RT navigation applications seems unrealistic.

Recently, a novel approach called the Proper Generalized Decomposition (PGD) has appeared to approximate the solutions of non-linear convex variational problems [21]. It is a new paradigm for solving classical problems in high-dimensional spaces [22,23]. In the PGD framework, the resulting model is solved once in order to obtain all the solutions for every possible value of the parameters defined in a specific domain, that is, a sort of computational vademecum. It could also be seen as a handbook containing all the possible solutions of a variational equation for any value of the parameters. Many challenging problems can be efficiently cast into this framework, and it opens new possibilities to solve problems with strategies not envisioned until now.

In the field of path planning, the goodness of having a precomputed solution when the obstacles and the goal are fixed was already stated in [16]. In the 1990s, the computation of the streamline maps for all the possible combinations of start/goal positions was not feasible with the available tools. Fortunately, this situation has now changed with the appearance of the PGD.

The goal of the present paper is to experimentally validate the PGD as a new alternative for RT mobile robot navigation using potential harmonic functions. Previous work was presented in [24] with local minima still present in the approximate solution due to the interaction of start and target positions, modelled as Gaussian functions. In the present work, cell-centred and staggered meshes are used to overcome this problem and a free-of-deadlocks solution is reached. To demonstrate the capabilities of the proposed approach, a harmonic potential field based on the flow theory is used in simulated complex environments as well as in RT navigation experiments with a LEGO^®^MINDSTORMS robot in environments with static obstacles and variable start and goal robot configurations. A LEGO^®^MINDSTORMS robot was also selected as the experimental platform because it is a commonly used device in teaching social robotics interactions [9]. This paper is organized as follows: Section 2 first details the potential flow theory to obtain harmonic functions by means of the Poisson equation. Additionally, the PGD-Vademecum solution for the mobile robot path planning introduced in our previous work [24] is revisited, and the restrictions required for the matrices to guarantee a free-of-deadlocks solution are defined. Section 3 presents the application of the PGD-Vademecum in simulated complex environments such as an L-Shaped corridor and a bug trap. Section 4 shows real experiments using a LEGO^®^MINDSTORMS robot in a square map with and without static obstacles. In Section 5, the computational properties of the PGD-based framework are disclosed. Finally, Section 6 draws conclusions and future works.

## 2. Previous Knowledge

### 2.1. Potential Flow Theory

Path planning based on the potential flow theory has been used in the literature over the last few years [12,14,16,17,18,19,20,25,26,27,28,29,30,31,32], focused mainly on the resolution of the Poisson equation. First of all, let us introduce the underlying mathematical model that describes a potential flow, where the velocity υ verifies

(1) ▽×υ=0,

and hence it is the gradient of a scalar potential function *u*, i.e., υ=−∇u. Then, assuming incompressible flow, i.e.,

(2) ∇·υ=0,

it results

(3) Δu=0.

Let us introduce a localized source (respectively, sink) modelled by a Dirac term $\delta_{S}$ (respectively, $-\delta_{T}$) on the right-hand side of (3). The velocity of the fluid is now the solution of the Poisson equation with source term $f=\delta_{S}-\delta_{T},$ that is,

(4) Δu=f.

Equation (4) has an infinite number of solutions. In order to solve it, appropriate boundary conditions must be introduced.

In what follows, Neuman boundary conditions compatible with the incompressibility constraint are enforced on the boundary Γ

(5) ∇u·n=q.

The resolution of the Poisson equation under these conditions produces a potential field from the starting point *S* to the target point *T*, without deadlocks [18]. Unfortunately, there is no analytical solution for this equation in the general case, and numerical techniques must be used. In this sense, the PGD technique, introduced in the next section, overcomes some of the drawbacks of common numerical methods allowing real-time performance.

### 2.2. Source Term Definition

Consider the functions $g_{S}:\Omega_{\underline{X}}\times \Omega_{\underline{S}}\to R$ and $g_{T}:\Omega_{\underline{X}}\times \Omega_{\underline{T}}\to R$ as 2D Gaussian density distributions centred in the start $\underline{S}=\left(s_{1},s_{2}\right)\in \Omega_{\underline{S}}$ and target configurations $\underline{T}=\left(t_{1},t_{2}\right)\in \Omega_{\underline{T}}$, respectively. Both functions are assumed to have equal variance given by parameter r>0,r∈R. More precisely, we can write $g_{S}=g_{S}\left(\left(x,y\right);\left(s_{1},s_{2}\right),r\right)={\left(2\pi r\right)}^{-1}e^{-\frac{1}{2r}\left({\left(x-s_{1}\right)}^{2}+{\left(y-s_{2}\right)}^{2}\right)},$ $g_{T}=g_{T}\left(\left(x,y\right);\left(t_{1},t_{2}\right),r\right)={\left(2\pi r\right)}^{-1}e^{-\frac{1}{2r}\left({\left(x-t_{1}\right)}^{2}+{\left(y-t_{2}\right)}^{2}\right)}$ and hence $\Omega_{\underline{X}}=\Omega_{x}\times \Omega_{y}$, $\Omega_{\underline{S}}=\Omega_{s}\times \Omega_{r}$ and $\Omega_{\underline{T}}=\Omega_{t}\times \Omega_{r}.$ Here, $\Omega_{\underline{X}}=\Omega_{\underline{S}}=\Omega_{\underline{T}}\subset R^{2}$.

In the context of path planning, the physical meaning of this model relies on the fact that uncertainty is always present in the vehicle position computation in the form of measurement noise and/or process noise. Both types of noise are usually modelled by Gaussian functions. Geometrically, in 2D, the standard deviations of the Gaussian functions define the radii of the uncertainty ellipse around the robot position. If the robot is holonomic (equal variance in X and Y positions), the ellipse turns into a circumference. If spherical bounding volumes are used to account for the vehicle size, in the case of an omnidirectional vehicle, these volumes can be transformed into an increase in the variance of these functions and an uncertainty bounding volume can be defined, as in [33], including the uncertainty area due to noise as well as the robot volume (radius of the bounding sphere). Additionally, a security radius can also be considered in this model in the context of minimum distance from walls.

Let us assume that the source term *f* in Equation (4) is non-uniform, that is, $f=g_{S}-g_{T}$ when $\left(x,y\right)\in \Omega_{\underline{X}}$ and zero otherwise. Then, the Poisson equation is now

$$-\Delta u=g_{S}-g_{T}$$

for given functions $g_{S}$ and $g_{T}$. Once discretized, a separated form of this equation is obtained in (6) as a function of the map, the start and target configurations:

(6) $$\begin{array}{cc} -\Delta u(\underline{X},\underline{S},\underline{T}) & =f(\underline{X},\underline{S},\underline{T}) \end{array}$$

### 2.3. A PGD-Vademecum Solution

The PGD-vademecum is generated considering that the solution of the potential field *u* solving (4) and (5) can be constructed as a finite sum of terms, each one consisting of the product of three functions: a function *R* of the environment $\underline{X}$, a function *W* of the start configuration $\underline{S}$ and a function *K* of the target or goal configuration $\underline{T}$:

(7) $$u^{n-1}\left(\underline{X},\underline{S},\underline{T}\right)=\sum_{i=1}^{n-1}R_{i}\left(\underline{X}\right)\cdot W_{i}\left(\underline{S}\right)\cdot K_{i}\left(\underline{T}\right)$$

and where the enrichment step is given by

(8) $$u^{n}=u^{n-1}+R\left(\underline{X}\right)\cdot W\left(\underline{S}\right)\cdot K\left(\underline{T}\right).$$

The key point is to find a rank-one function

(9) $$\begin{array}{c} R\left(\underline{X}\right)\cdot W\left(\underline{S}\right)\cdot K\left(\underline{T}\right):=\\ R\left(x,y\right)\cdot W\left(s_{1},s_{2}\right)\cdot K\left(t_{1},t_{2}\right) \end{array}$$

satisfying

(10) $$\int_{\Omega_{\underline{X}}\times \Omega_{\underline{S}}\times \Omega_{\underline{T}}}u^{*}\cdot \left(\Delta u^{n}-f\right)\;d\Omega_{\underline{X},\underline{S},\underline{T}}=0$$

for all test functions $u^{*}$ in the linear space of functions

(11) $$\begin{array}{c} R^{*}\left(\underline{X}\right)\cdot W\left(\underline{S}\right)\cdot K\left(\underline{T}\right)+R\left(\underline{X}\right)\cdot W^{*}\left(\underline{S}\right)\cdot K\left(\underline{T}\right)\\ +R\left(\underline{X}\right)\cdot W\left(\underline{S}\right)\cdot K^{*}\left(\underline{T}\right). \end{array}$$

There are some techniques to measure the error in the approximation versus the number of PGD terms (*n*). One of the most appropriate error estimators is the $L^{2}\left(\Omega_{\underline{X}}\times \Omega_{\underline{S}}\times \Omega_{\underline{T}}\right)$-residual R(n) obtained by inserting the PGD-vademecum approximation into the Poisson Equation (6) and calculating the residual (10), that is

(12) $$R\left(n\right)=\int_{\Omega_{\underline{X}}\times \Omega_{\underline{S}}\times \Omega_{\underline{T}}}\left(\Delta u^{n}-f\right)\cdot \left(\Delta u^{n}-f\right)\;d\Omega_{\underline{X},\underline{S},\underline{T}}.$$

One of the most important properties of the PGD is that the first terms store more relevant information than the last terms, see [24].

### 2.4. Meshing Constraints to Guarantee Free-of-Deadlocks Solutions

In our previous work [24], the PGD was first introduced as an approach capable of using potential fields methods based on harmonic functions for real-time navigation. However, since the PGD is an approximation method, it did not guarantee that the resulting approximate solution was free of local minima. In this work, this problem has been solved and the resulting PGD solution is free of deadlocks as explained in [34] and summarized here:

- The mesh of $\underline{X}$ must be cell-centred with the $\underline{S}$ and $\underline{T}$ meshes. This means that the physical positions of the nodes $\underline{S}$ and $\underline{T}$ must also exist in the $\underline{X}$ mesh. It is obvious as all the robot origins and destinations must appear on the map. In addition, the dimensions of $\underline{S}$ and $\underline{T}$ can be much smaller than the dimension of $\underline{X}$, since all the start (and goal) positions could be separated a moderated distance (0.5 m, for example) while the navigation map must be accurate (with separation between nodes of a few cm).
- The mesh of $\underline{S}$ must be staggered with the $\underline{T}$ mesh.

## 3. Simulated Validation. Construction of the PGD-Vademecum in Complex and Real Environments

**Figure 1:**
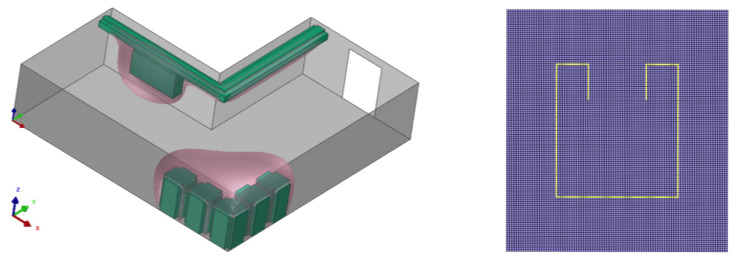
Complex and real environments for navigation: (**left**) L-shaped corridor in a plant; (**right**) bug trap planning environment.

**Figure 2:**
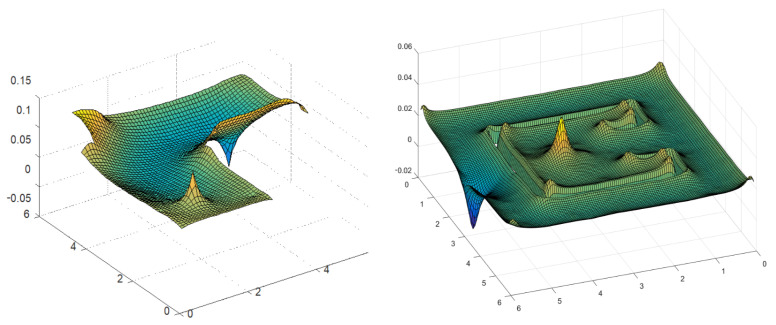
PGD reconstruction (potential field mesh) for two arbitrary start and goal locations in a (**left**) L-shaped corridor and (**right**) a bug trap planning environment.

In this section, the construction of a PGD-vademecum for path planning using the Poisson equation is explained for two complex and real environments depicted in Figure 1. The environment on the left is an L-shaped corridor of a plant with two zones (in pink) that should be avoided during the robot navigation. The picture on the right represents a bug trap planning environment used for benchmarking in robot motion planning. In both cases, the walls have been modelled with Neumann boundary conditions. In the case of the L-shaped corridor, the dangerous zones have been modelled with stronger Neumann boundary conditions than those employed for the walls. In the first example, the corridor is 6 m long, the $\underline{X}$ mesh has $N_{x}=N_{y}=60$ nodes while the $\underline{S}$ and $\underline{T}$ meshes have a dimension of $N_{x}=N_{y}=6$ nodes. In the second example, the environment is a 6 × 6 m square where the $\underline{X}$ mesh has $N_{x}=N_{y}=120$ nodes, and the $\underline{S}$ and $\underline{T}$ meshes have $N_{x}=N_{y}=12$ nodes. Figure 2 represents the PGD reconstructions for both cases using two arbitrary start and goal positions with n=300 PGD terms. These simulations were developed in MATLAB using an HP laptop with a CPU Intel CORE i5, 4 GB RAM memory.

## 4. Experimental Validation. PGD-Vademecum in a Lego ^®^Mindstorms

**Figure 3:**
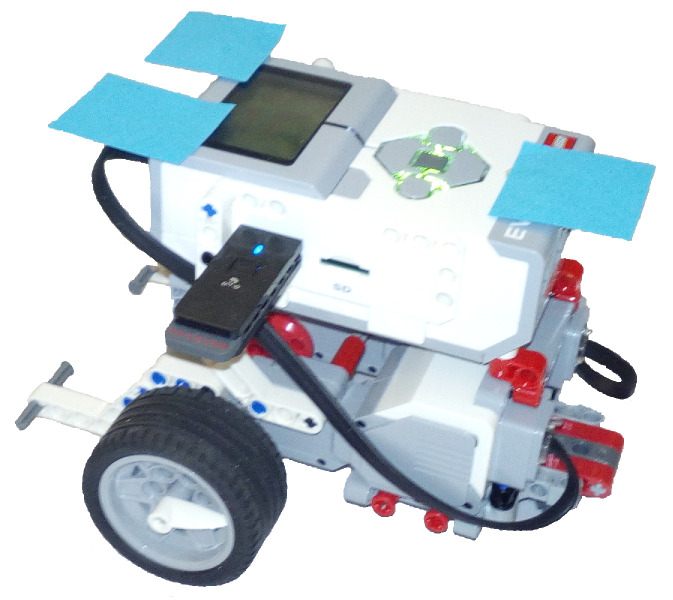
Experimental LEGO robot.

In order to test the benefits of the PGD framework, the PGD-vademecum must be programmed in a real mobile robot with restricted computational and memory capabilities. In this sense, a LEGO^®^MINDSTORMS Education EV3 Core Set has been selected. The Core Set includes 541 elements usually employed for teaching science, technology, engineering, mathematics, and computer science to children. The Core Set comes in a sturdy storage bin with a sorting tray for easy classroom management and includes three servomotors and a computer-in-a-brick that makes it possible to control motors and collect data from sensors. The brick has a LINUX operating system with an ARM controller that runs at 300 MHz with a 64 MB RAM and 16 MB flash memory. These computational and memory capabilities are far from those of the recent CPUs. An omnidirectional robot has been built with the EV3 Brick, two servomotors and some common LEGO^®^MINDSTORMS pieces. The robot is shown in Figure 3.

**Figure 4:**
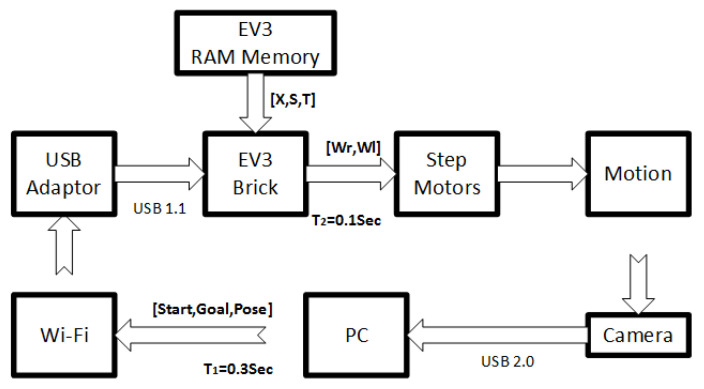
Block diagram of the Lego^®^ vehicle experimental setup.

The EV3 Brick can be directly programmed in C++ but a Matlab^®^ compiler with the LEGO^®^EV3 library for Simulink^®^ is used instead. The wireless communication between the EV3 Brick and the PC is established by Wi-Fi. The block diagram of the experimental setup is depicted in Figure 4. A webcam with 1280×720 pixels is used to sense the 1.5 × 1.5 m map where the vehicle moves. The camera is connected to a desktop PC, in particular, an HP laptop with Intel CORE i5, 4 GB RAM memory. The PC computes, on the one hand, the actual pose(x,y,θ) of the LEGO^®^ vehicle from the position of three blue squares attached to the top of the vehicle and, on the other hand, the target position $\left(X_{g},Y_{g}\right)$ indicated by a red plastic disk. This information is sent to the LEGO^®^ brick in real-time. Detailed explanations about the implementation setup can be found in the authors’ previous work [35].

The EV3 Brick stores the compiled code that controls the vehicle as well as the PGD-Vademecum matrices $\left(\underline{X},\underline{S},\underline{T}\right)$, denoted by (X,S,T) in the RAM memory. Each of the above matrices has a particular $N_{x}\times N_{y}$-size depending on the size of the *x* and *y* meshes of $\underline{X},$$\underline{S}$ and $\underline{T}.$

Given the desired wheels velocities, a PID is used to control the vehicle trajectory. These velocities are computed as explained in the following subsections.

### 4.1. PGD-Vademecum to Compute the Wheels Velocities

**Figure 5:**
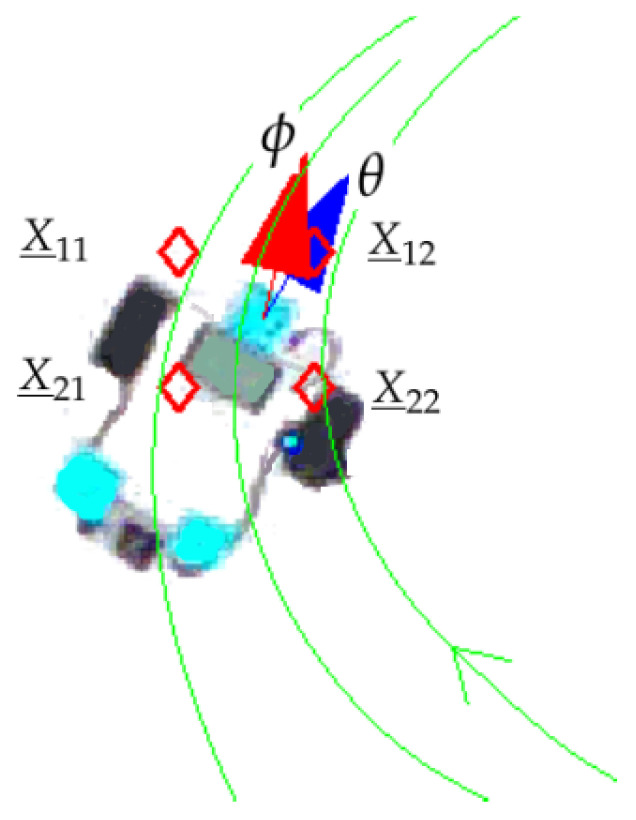
Coordinates of neighbour nodes in a particular mobile robot position.

One of the advantages of the PGD-vademecum in contrast to FEM simulation techniques used in [18] is that it allows the reconstruction of single mesh nodes in each algorithm execution *k* and for any kind of parameter combination. In the case of a robotics application, this property implies the possibility of computing the mobile robot path in a small portion of the map for any combination of the start and goal configurations $\underline{S}$, $\underline{T}$. The so-called Region Of Interest (ROI) is composed of the four neighbouring nodes of a particular vehicle position $\underline{X}=\left(x,y\right)$ and can be described by a 2×2 block matrix $ROI(\underline{X})$ as

(13) $$ROI\left(\underline{X}\right)=[\begin{array}{cc} {\underline{X}}_{11} & {\underline{X}}_{12}\\ {\underline{X}}_{21} & {\underline{X}}_{22} \end{array}]$$

where ${\underline{X}}_{11}=\left(x_{1},y_{1}\right)$, ${\underline{X}}_{12}=\left(x_{1},y_{2}\right)$, ${\underline{X}}_{21}=\left(x_{2},y_{1}\right)$ and ${\underline{X}}_{22}=\left(x_{2},y_{2}\right)$ are the coordinates of the neighbouring nodes of $\underline{X}$, represented as red rhombuses in Figure 5.

Selecting $\underline{S}=\left(x_{S},y_{S}\right)$ as the start node position and $\underline{T}=\left(x_{T},y_{T}\right)$ as the goal node position, the ROI-Poisson approximate solution at $\underline{X}$ can be simply computed using the PGD-vademecum solution *u* as

(14) $$u_{ROI}\left(\underline{X},\underline{S},\underline{T}\right)=\left[\begin{array}{cc} u({\underline{X}}_{11},\underline{S},\underline{T}) & u({\underline{X}}_{12},\underline{S},\underline{T})\\ u({\underline{X}}_{21},\underline{S},\underline{T}) & u({\underline{X}}_{22},\underline{S},\underline{T}) \end{array}\right].$$

The ROI velocity field matrix can be computed as indicated in Equation (15), where $V_{x_{ROI}},V_{y_{ROI}}$ are 2×2 matrices with the velocity field for each node.

(15) $$\begin{array}{cc} V_{x_{ROI}}=\frac{\partial u_{ROI}}{\partial x}\left(\underline{X},\underline{S},\underline{T}\right) & =\left[\begin{array}{cc} \frac{\partial u}{\partial x}\left({\underline{X}}_{11},\underline{S},\underline{T}\right) & \frac{\partial u}{\partial x}\left({\underline{X}}_{12},\underline{S},\underline{T}\right)\\ \frac{\partial u}{\partial x}\left({\underline{X}}_{21},\underline{S},\underline{T}\right) & \frac{\partial u}{\partial x}\left({\underline{X}}_{22},\underline{S},\underline{T}\right) \end{array}\right]\\ \; & \\ V_{y_{ROI}}=\frac{\partial u_{ROI}}{\partial y}\left(\underline{X},\underline{S},\underline{T}\right) & =\left[\begin{array}{cc} \frac{\partial u}{\partial y}\left({\underline{X}}_{11},\underline{S},\underline{T}\right) & \frac{\partial u}{\partial y}\left({\underline{X}}_{12},\underline{S},\underline{T}\right)\\ \frac{\partial u}{\partial y}\left({\underline{X}}_{21},\underline{S},\underline{T}\right) & \frac{\partial u}{\partial y}\left({\underline{X}}_{22},\underline{S},\underline{T}\right) \end{array}\right] \end{array}$$

The velocity vector in the mobile robot position (x,y) at step *k* can be computed by means of a bi-linear interpolation technique as

(16) $$\upsilon_{x}\left(\underline{X}\right)=\left(\begin{array}{cc} 1-x^{*} & x^{*} \end{array}\right)V_{x_{ROI}}\left(\begin{array}{c} 1-y^{*}\\ y^{*} \end{array}\right)$$

(17) $$\upsilon_{y}\left(\underline{X}\right)=\left(\begin{array}{cc} 1-x^{*} & x^{*} \end{array}\right)V_{y_{ROI}}\left(\begin{array}{c} 1-y^{*}\\ y^{*} \end{array}\right)$$

where $\left(x^{*},y^{*}\right)$ are the normalized distances:

(18) $$x^{*}=\frac{x_{2}-x}{x_{2}-x_{1}},y^{*}=\frac{y_{2}-y}{y_{2}-y_{1}}$$

The resulting vector must be re-scaled to guarantee that the vehicle follows the streamline at constant velocity by taking

(19) $$\begin{array}{cc} \phi & =\arctan\left(\frac{\upsilon_{y}\left(\underline{X}\right)}{\upsilon_{x}\left(\underline{X}\right)}\right)\\ \; & \\ v & =\upsilon_{0}, \end{array}$$

where $\upsilon_{0}$ represents the constant mobile robot’s velocity. Assuming that $\underline{X}$ is the vehicle position at time instant *t*, i.e., $\left(x\left(t\right),y\left(t\right)\right)=\underline{X}$, then its position at t+Δt (previously fixed time step Δt), denoted by (x(t+Δt),y(t+Δt)), is given by

(20) $$x\left(t+\Delta t\right)=x\left(t\right)+\upsilon_{0}\cdot \cos\left(\phi \right)\Delta t,$$

(21) $$y\left(t+\Delta t\right)=y\left(t\right)+\upsilon_{0}\cdot \sin\left(\phi \right)\Delta t,$$

and hence the displacement on each axis between instants *t* and t+Δ is given by:

(22) $$\Delta x=\upsilon_{0}\cdot \cos\left(\phi \right)\cdot \Delta t,$$

(23) $$\Delta y=\upsilon_{0}\cdot \sin\left(\phi \right)\cdot \Delta t.$$

Finally, the angular displacement on the wheels $\left(\Delta \alpha_{r},\Delta \alpha_{l}\right)$ between *t* and t+Δ are computed, as usual, by means of inverse kinematics:

(24) $$\left(\begin{array}{cc} \Delta \alpha_{r} & \Delta \alpha_{l} \end{array}\right)=\left(\begin{array}{ccc} \frac{\cos(\phi )}{R} & \frac{\sin(\phi )}{R} & \frac{L}{R}\\ \frac{\cos(\phi )}{R} & \frac{\sin(\phi )}{R} & -\frac{L}{R} \end{array}\right)\cdot \left(\begin{array}{c} \Delta x\\ \Delta y\\ \Delta \phi \end{array}\right)$$

where *R* is the wheel radius, *L* is the wheel separation and Δϕ=ϕ−θ. Figure 5 displays a snapshot of a particular robot pose (x,y,θ). The blue arrow shows the robot orientation θ, and the red arrow shows the computed reference for the next step, k+1, by means of the ROI. Green curves are the continuous streamlines derived from the potential field.

### 4.2. Experimental Tests

Three tests have been developed with the LEGO^®^ mobile robot in the 1.5 × 1.5 m square environment and are explained in the following paragraphs. In them, a red plastic disk is used as a random goal.

1. **Test 1: Point to Point.**
  This test shows that the PGD-vademecum could cover all the possible start and goal combinations ${\left(N_{x}\times N_{y}\right)}^{2}$ for the vehicle position. The test procedure is as follows: the red disk is thrown to the scene, falling into an arbitrary position, and the vehicle must reach the red disk (goal); once the robot reaches the goal, the red disk is thrown to another arbitrary position.
2. **Test 2: Dynamic Goal.**
  This test demonstrates that the robot does not need additional computation when the goal changes before the robot reaches it, unlike in [18] where, if the goal changes while the robot is following a particular streamline, a new FEM simulation must be executed and the robot has to wait for the new FEM simulation solution. The test procedure is the following: the red disk is thrown to the scene falling into an arbitrary position; the red disk (goal) changes in real-time (due to external causes) forcing the robot to adjust its target while navigating the environment.
3. **Test 3: Perturbations.**
  This tests demonstrates the robustness of the potential field approach for each particular PGD-vademecum solution. For that purpose, the robot is perturbed, changing its pose before reaching the goal. The procedure of this test is as follows: the red disk is thrown to the scene falling into an arbitrary position; the robot pose is manually changed to another arbitrary pose, forcing it to recompute its trajectory in real-time.

These three tests are performed in two modalities:

1. *Modality A: Without static obstacles.*
  In this modality, the robot navigates a square environment of 1.5 × 1.5 m discretized with 20×20 nodes. The precomputed PGD-Vademecum has the following parameters: r=0.7, n=200 and q=0.06.
2. *Modality B: With a static obstacle.*
  In this modality, the robot navigates a 1.5 m × 1.5 m square with a square static obstacle of 0.3 × 0.3 m located at the centre of the environment. This would be a common situation for an autonomous robot moving in a house, a parking lot, a field with trees, etc., where the map contains static obstacles, and the robot has to skirt them. The environment is also discretized with 20×20 nodes. The precomputed PGD-vademecum has the following parameters: r=0.7 and n=200 and q=1. The generation of static obstacles only involves the assignation of boundary conditions to the nodes belonging to each static obstacle. It allows the construction of any configuration map.

**Figure 6:**
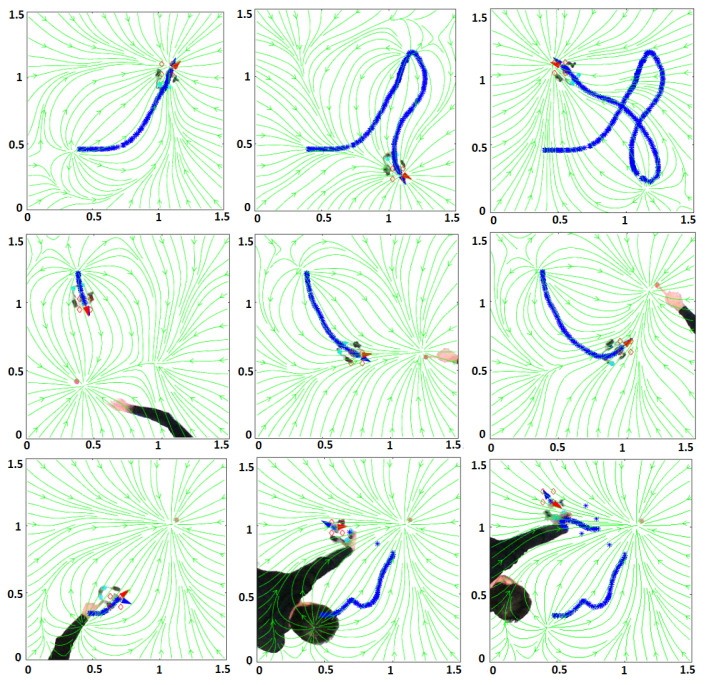
Experimental tests with a LEGO robot. Rows 1, 2, 3 for tests 1A, 2A, 3A.

**Figure 7:**
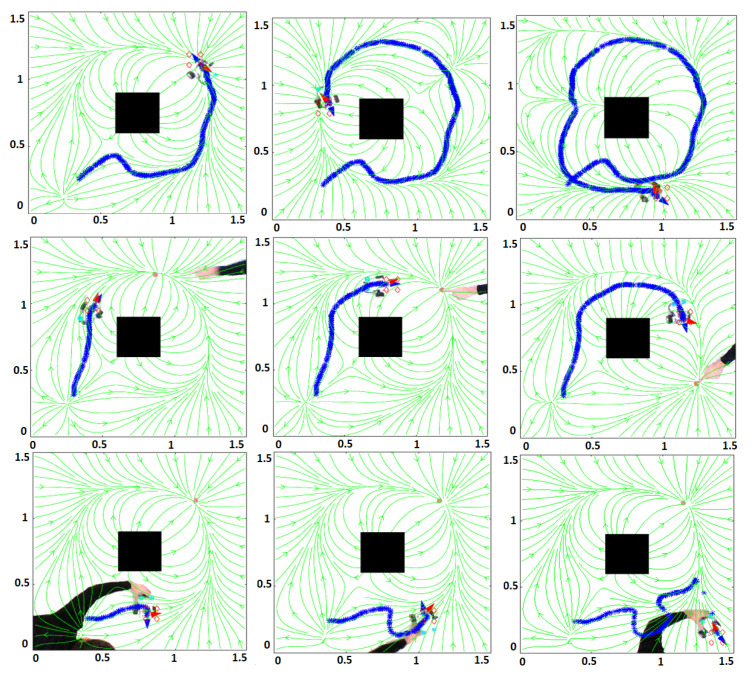
Experimental tests with a LEGO robot. Rows 1, 2, 3 for tests 1B, 2B, 3B.

Figure 6 shows some snapshots of the experiments carried out for tests 1A, 2A, 3A. Figure 7 shows some snapshots of the experiments performed for tests 1B, 2B, 3B. The robot velocity in all the tests was v=0.1 m/s. In [36], a video with all the tests is shown.

## 5. Time and Memory Complexity of the Method

**Figure 8:**
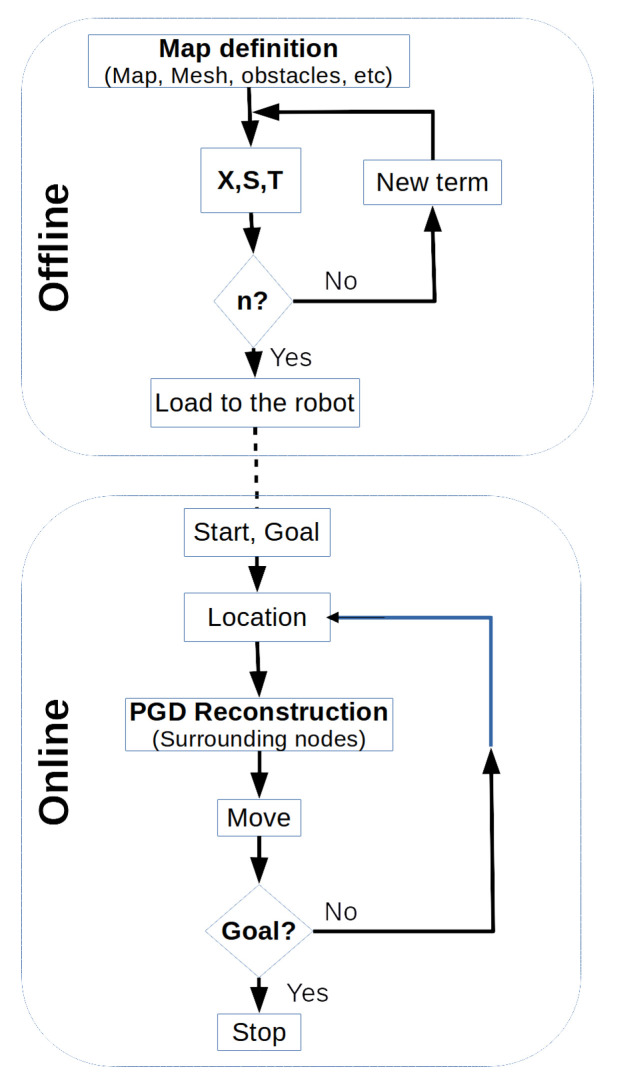
Flowchart of the two phases (off-line and on-line) involved in the PGD-based path planning method.

**Figure 9:**
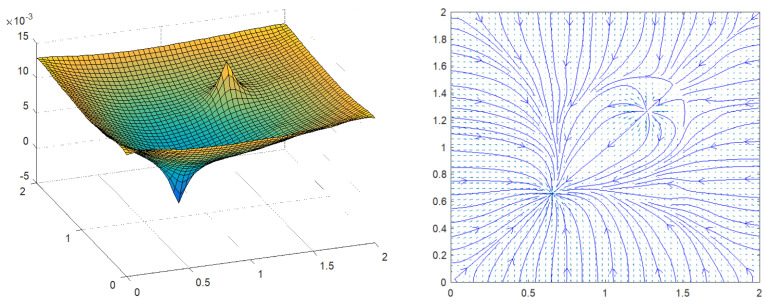
Numerical examples for two arbitrary $\underline{S},\underline{T}$ locations in two complex environments.

The method presented in this work consists of two phases: an off-line phase and an on-line phase, as depicted in the flowchart of Figure 8. In the off-line phase, the aim is to obtain an approximation of the Poisson equation through the PGD method. This stage provides a solution in the form of a system of uncoupled matrices. These matrices can be easily combined in the on-line phase in order to reconstruct the required solution in an extremely efficient manner, as it only implies the computation of a set of sums and products. The implementation presented in this paper uses the fixed point iteration method to calculate the uncoupled matrices X,S and *T*. In the numerical example presented in Figure 9, the off-line computational cost of calculating each of the PGD terms is 1.3 s on a Dell Notebook, with an ^®^Intel Core i7-8550U processor at 1.80 Ghz and 16 GB RAM. The space occupied in memory by the resulting matrices using $N_{x}=N_{y}=50$ for matrix *X* and $N_{x}=N_{y}=5$ for matrices S,T is 2.9 MB using simple precision floating-point numbers. An extrapolation of the results of B type tests can be made for the map of a real small village, such as 1000 × 1000 m. In this case, selecting a resolution of 0.5 m, the entire map would have a size of 5.8 GB.

Once the off-line process has finished, the resulting *X*, *S* and *T* matrices are stored in the robot’s memory for it to navigate the map using the approximate solution obtained through the PGD. The process is performed as shown in Figure 8: given an initial vehicle position and any destination within the map, in every algorithm iteration only a portion of the path around the vehicle location is reconstructed, the ROI, composed by the surrounding nodes of the vehicle position (Figure 5). This calculation is modelled in Equation (13), taking into account the tensor representation of *u* by (25), and entails the realization of eight products and n sums. If the number of PGD terms is, for instance, n=300, it would imply a total of 2400 floating-point operations. In a modest desktop computer with a Pentium 4 or Athlon 64-type processor, which typically operates faster than 3 GHz and has a computational performance in the range of a few GFLOPS, this on-line calculation would take 2.3 ms considering one GFLOPS performance. Furthermore, if an Intel ^®^Core i7 microprocessor were used and taking into account that it operates in the range of TFLOPS, the on-line phase would take a few μs, a negligible processing time. Therefore, the computational time of the path reconstruction using the PGD solution is really low, nearly constant O(1) and invariant with respect to the size of the map and the selected map resolution.

(25) $$u\left(\underline{X},\underline{S},\underline{T}\right)=\sum_{i=1}^{n}R_{i}\left(\underline{X}\right)W_{i}\left(\underline{S}\right)K_{i}\left(\underline{T}\right)$$

**Figure 10:**
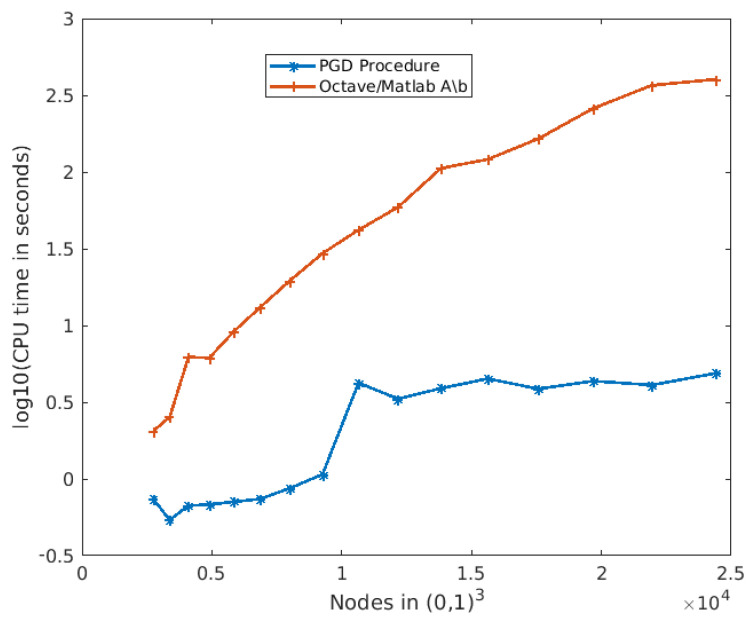
Comparison made in [37] in terms of CPU time between the PGD method and the traditional A\b Matlab-Octave method for the resolution of the Poisson equation as a function of the number of nodes in the discretization of the equation.

Regarding the off-line phase, specific details about the method of convergence, computational time, complexity, etc., can be found in the previous authors’ work [37], where numerical examples are provided in order to describe the relationship between the PGD (also called the Greedy Rank-One Update algorithm) and the FEM for high-dimensional partial differential equations based on the tensor product of one-dimensional bases. A comparison between the PGD method and the standard linear system solver of Matlab-Octave A\b for the resolution of the Poisson equation is provided in that paper and is also depicted in Figure 10, where the evolution of the CPU time in seconds for both algorithms as a function of the number of nodes used in the discretization of the Poisson equation is shown for a high-performance computer ^®^Intel Xeon Gold 6130 CPU at 2.1 GHz and 128 GB RAM (64-bit 16-core x86 multi-socket high performance server microprocessor). This illustration helps to understand at a glance the importance of this method as a numerical solver. It is worth noting that with the traditional method, A\b it is necessary to solve the equation for the whole map in real-time in every algorithm execution and, therefore, the curse of dimensionality appears when the number of nodes increases (big maps). Nonetheless, with the PGD method, each term and node are decoupled, and it is possible to reconstruct only a simple node or a region of interest as a sum of multiplications, with the computational time independent of the size and resolution of the map.

Finally, Figure 10 allows the comparison between the proposed method and any other grid-based planning method for different grid sizes. For instance, in [38], the A* algorithm is used for real-time motion planning on a 30 × 45 m map discretized using a high resolution grid of 150 × 225 nodes. The entire path planned by the motion planning algorithm contains 2–3 s of commands and assumes an 80 ms processing time (although it can exceed 450 ms if the goal is unachievable). Using the PGD-based framework with a similar but square environment of 150 × 150 nodes (22,500 nodes) and according to Figure 10, the off-line phase would take 14 h in a high-performance computer resulting in the solution of the equation for every start and goal vehicle position. The on-line phase would take negligible time, as the reconstruction is carried out at every time cycle with the same ROI size.

## 6. Conclusions and Future Work

The present paper validates the applicability of the numerical technique known as PGD-vademecum to global path planning for mobile robots using harmonic functions. This method produces, for a predefined map, a vademecum containing all the possible vehicle paths for any combination of the start and goal configurations within the map. The PGD-vademecum is computed off-line and reconstructed on-line for any particular combination of the start and goal configurations. It is really fast as its formulation is a simple sum of products. In fact, in RT applications, only the surrounding nodes of the vehicle position need to be reconstructed every execution cycle. As a consequence, the computational costs are nearly negligible, which allows its implementation even in robots with restricted computational capabilities, such as a LEGO^®^EV3 toy. The resulting paths are based on the Laplace/Poisson equation and, therefore, are free of deadlocks. This property makes it a promising technique to solve the piano mover’s problem for any type of robots, such as social robots, because many parameters can be introduced in the PGD-Vademecum as extra coordinates, for instance, parameters related to different kinematic or dynamic robot models or even dynamic obstacles. In this sense, the immediate future work is related to the inclusion of mobile obstacles in the environment. There are various possibilities to take dynamic obstacles into account, such as the one studied in [39], where the disturbance in conductivity is used to model the presence of a moving obstacle, and the location of that conductivity disturbance is included as an additional parameter in the PGD formulation. Other future works imply the consideration of kinematic models for non-holonomic robots in the PGD framework and its use in solving the motion planning problem for unmanned aerial vehicles.
